# A case of eosinophilic granulomatous polyangiitis with concurrent central and peripheral nervous system involvement

**DOI:** 10.1093/omcr/omad067

**Published:** 2023-07-18

**Authors:** Anusha Challa, Sai Sirisha, Harsh Khandelia, Mihir Parekh, Anuja Patil, Sita Jayalakshmi

**Affiliations:** Department of Neurology, Krishna Institute of Medical Sciences, Secunderabad, India; Department of Neurology, Krishna Institute of Medical Sciences, Secunderabad, India; Department of Critical Medicine, Krishna Institute of Medical Sciences, Secunderabad, India; Department of Neurology, Krishna Institute of Medical Sciences, Secunderabad, India; Department of Neurology, Krishna Institute of Medical Sciences, Secunderabad, India; Department of Neurology, Krishna Institute of Medical Sciences, Secunderabad, India

## Abstract

Eosinophilic granulomatous polyangiitis (EGPA) like other antineutrophil cytoplasmic antibody (ANCA)-associated vasculitis has multisystemic involvement. It commonly manifests with prodromal pulmonary involvement as asthma, chronic sinusitis followed by systemic vasculitic complications associated with blood and tissue eosinophilia. Central nervous system manifestations at presentation are uncommon compared with peripheral nervous system involvement. Vasculitic neuropathy in EGPA commonly presents as mononeuritis multiplex but rarely as polyradiculopathy. Late onset EGPA often presents with systemic involvement, and early diagnosis is a key to prevent further complications. The neuropathy in late onset EGPA is often refractory to immunosuppression and corticosteroids treatment. We report a case of EGPA with late onset asthma presenting with acute infarct and demyelinating polyradiculoneuropathy that progressed with bulbar paralysis and profound dysautonomia. This illustrates simultaneous involvement of central and peripheral nervous system with EGPA. Autonomic dysfunction can occur in patients of EGPA with multisystem involvement, which may predict severe complications.

## INTRODUCTION

Eosinophilic granulomatosis with polyangiitis (EGPA), formerly known as Churg–Strauss syndrome (CSS), is a small-vessel antineutrophil cytoplasmic antibody (ANCA)-associated vasculitis [1]. It is characterized by initial prodromal phase with asthma and chronic rhinitis, and subsequently, eosinophilic phase, with peripheral blood eosinophilia and eosinophilic infiltration of organs like skin, lung and gastrointestinal tract. The final phase is characterized by systemic vasculitis of the small and medium vessels. Peripheral nervous system involvement was reported in 55–72%, whereas central nervous system involvement is uncommon (5–9%) [2]. Neurological manifestations at presentation are rare. Late presentation in predicts poor prognosis with multiple system involvement [3].

## CASE SUMMARY

A 61-year-old woman presented with sudden onset weakness in right upper and lower limbs for 4 days. About 2 days before the symptom onset, she had developed painful paresthesias of bilateral distal upper and lower limbs. Her medical history revealed late onset asthma with frequent exacerbations treated with oral steroids, montelukast and salbutamol nebulization for past 6 years. Examination showed small scattered erythematous lesions with surface erosions on both lower limbs. She had right hemiparesis (Medical Research Council-MRC grade 3/5 in upper limb and 2/5 in lower limb) with right central facial palsy, diffuse areflexia with loss of vibration and proprioception in distal limbs. An MRI of the Brain showed left internal capsule lacunar infarct with normal angiography. 2DEchocardiography was normal. Lipid profile, thyroid, HbA1c and renal parameters were normal. She was started on antiplatelet, statins and initiated on regular limb physiotherapy.

**Figure 1:**
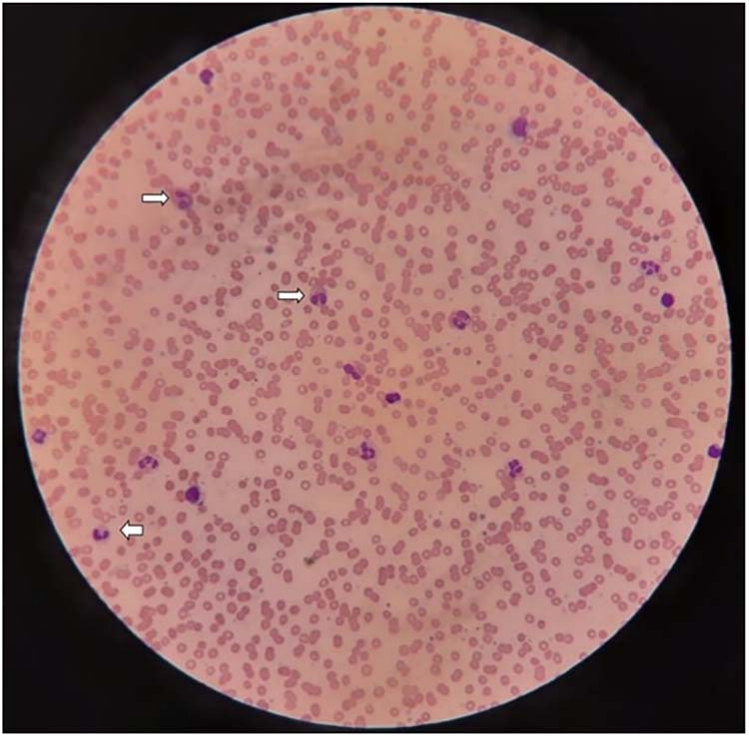
Leishman-stained section of peripheral smear; showing normocytic, normochromic RBC’s, moderate leukocytosis with severe eosinophilia (arrows) and adequate platelets with normal morphology (seen at ×20 magnification).

Over next 5 days, she developed progressive flaccid quadriplegia with bulbar weakness and bilateral facial palsy (MRC grade 0/5). Nerve conduction studies showed demyelinating polyradiculoneuropathy with temporal dispersion and prolonged F wave latencies and conduction block in right median nerve with reduced sensory nerve action potential amplitudes. Considering acquired demyelinating polyradiculoneuropathy, she was started on intravenous immunoglobulins (IVIG). Her peripheral blood smear showed significant eosinophilia of 39% (Fig. 1B). In view of asthma, peripheral eosinophilia, progressive polyradiculopathy and lacunar infarct, diagnosis of eosinophilic granulomatosis with polyangiitis was considered and pulse methyl-prednisolone was added.

**Figure 2:**
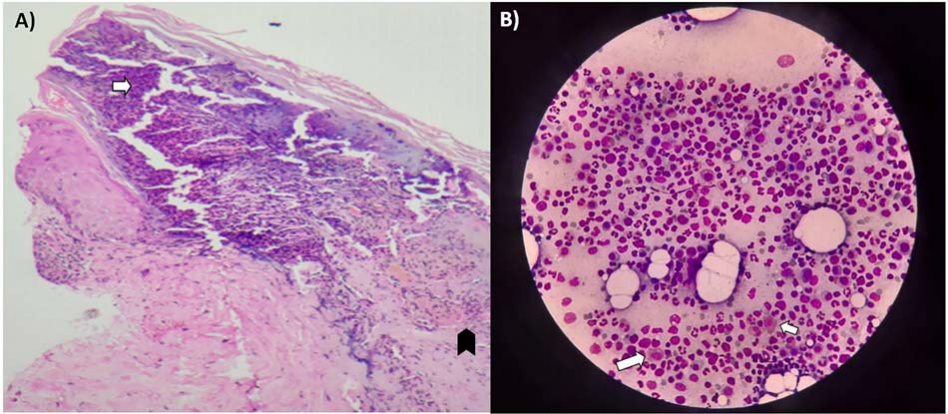
(**A**) Hematoxylin and eosin-stained section of a skin biopsy showing subepidermal inflammation (arrow) extending into superficial dermis with many eosinophils, perivascular leukocytoclastic deposits (dark arrow head) and absence of fibrinoid necrosis and granulomas suggestive of leukocytoclastic vasculitis (seen at ×20 magnification). (**B**) Leishman and Giemsa-stained section of Bone marrow smear showing adequate trilineage hematopoiesis, increased eosinophilic precursors (marked with arrows) without any blast cells or granulomas (seen at ×40 magnification).

**Figure 3:**
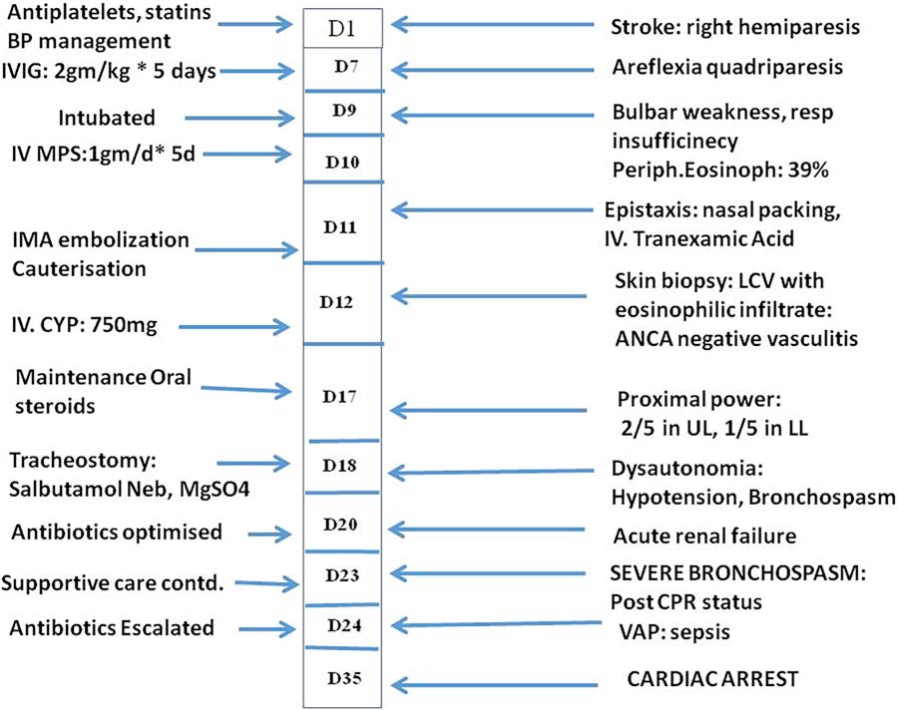
Clinical course from onset of symptoms. Abbreviations—IV, intravenous; MPS, methylprednisolone.

Despite IVIG, on Day 9, she developed respiratory weakness requiring ventilatory support. She developed spontaneous severe epistaxis refractory to local management with normal coagulation parameters and platelet count. Digital subtraction angiography localized source of bleeding from the inferior maxillary artery (IMA), which was then embolized. However, she still had mild bleeding from the local mucosal surface. So, local cauterization was done under general anesthesia. Meanwhile, her blood IgE level showed marked elevation (14 370 UI/ml). She had normal renal parameters, normal complement C3 and C4 levels, and anti-dsDNA, ANA profile, p ANCA and c ANCA, antigen-specific assays for myeloperoxidase and proteinase3 were negative. Abdominal ultrasound was normal, whereas HRCT chest revealed ground glass opacities in bilateral lower lobe suggestive of pulmonary infiltrates. Skin biopsy from the erythematous lesions showed leukocytoclastic vasculitis (LCV) with pericapillary eosinophilic infiltrates (Fig. 2A). A bone marrow biopsy was done, which showed adequate trilineage hematopoiesis with increased eosinophilic precursors without granulomas confirming the diagnosis of EGPA (Fig. 2B). Oral prednisolone was continued, and cyclophosphamide (CYP, 750 mg) was given. She had significant dysautonomia despite ongoing treatment with systolic blood pressure fluctuating between 90 and 170 mm Hg and intermittent bradycardia up to 40 beats per minutes. She showed mild improvement in strength of both upper limbs to 2/5 and lower limbs to 1/5 MRC grade and was weaned off ventilator support after tracheostomy. However, she continued to have dysautonomia with paroxysms of severe bronchospasm occurring spontaneously. She was given nebulized bronchodilators and steroids (Budesonide), oral prednisolone. During an episode of bronchospasm, she had cardiac arrest with asystole on Day 23 of her hospitalization. She was revived after cardio-pulmonary resuscitation and continued supportive care. Subsequently, she developed renal failure, ventilator-associated pneumonia (VAP) and sepsis and succumbed on Day 35. Her clinical course has been depicted in Fig. 3.

## DISCUSSION

As per the American College of Rheumatology 1990 criteria for the classification of EGPA, out of following six criteria—asthma, eosinophilia > 10%, mononeuropathy or polyneuropathy, non-fixed pulmonary infiltrates, paranasal sinus abnormality and extravascular eosinophils, four criteria are required for the diagnosis of EGPA with specificity of 99.7% [4].

The present case had late onset asthma and peripheral eosinophilia with simultaneous central and peripheral nervous system involvement in the form of a lacunar internal capsule infarct and demyelinating polyradiculoneuropathy. She had rapid progression, developed severe dysautonomia and multisystem involvement despite aggressive immunomodulation with fatal outcome.

Central or peripheral neurological manifestations as initial presentation in EGPA have been reported to occur as often as 62% [5]. Mononeuritis multiplex is considered the classical presentation and relatively more common than the presentation of acute symmetrical polyneuropathy [6]. The most common systemic feature reported in EGPA is asthma (91–100%), followed by ENT involvement (48–75%), peripheral neuropathy (55–72%), cutaneous features (40–52%) and less commonly central nervous system (5–9%) [2]. ANCA positivity was detected in 38% of cases and correlates to disease severity when present [2]. However, the occurrence of systemic complications is not different compared with those negative for ANCA.

Painful paresthesias have been described in almost 55% cases as sensory manifestation often predicting onset of motor deficits or systemic complications [7]. This may result from local vasculitis causing cutaneous and neuronal ischemia. Severe dysautonomia in our case could be the result of polyneuropathy involving the autonomic nervous system or because of a central mechanism. Dysrhythmias because of impaired vagal modulation of heart rate in the absence of structural heart disorders have been associated with EGPA even during disease remission [8].

Although EGPA manifests at younger age, late onset (60 years and above) cases are reported [3] and shown to have frequent systemic complications. Neuropathy resistant to immunosuppression and corticosteroids is more commonly noticed in elderly [3]. Multiple systemic causes (Table 1) may be associated with the risk of stroke and involvement of peripheral nervous system, albeit concurrent presentations are rare.

**Table 1:** Differential diagnoses for stroke with peripheral neuropathy.

Categories	Etiologies
Inflammatory	Systemic lupus erythematosus, sarcoidosis
Vascular	Systemic vasculitis: granulomatosis with polyangiitis, Sjogren’s syndrome, polyarteritis nodosa, CSS, cryoglobulinemic vasculitis
Metabolic	Diabetes mellitus, POEMS syndrome, systemic amyloidosis
Infections	HIV, hepatitis B and C, Sars-CoV-2
Toxic	Alcohol, cocaine abuse, lead intoxication
Inherited	Fabry’s disease

POEMS, polyneuropathy, organomegaly, endocrinopathy, monoclonal gammopathy and skin changes syndrome; HIV, human immunodeficiency; Sars-CoV-2, severe acute respiratory syndrome coronavirus 2.

Vasculitic neuropathy in Eosinophilic granulomatosis with polyangiitis may masquerade as acute inflammatory demyelinating polyneuropathy. Stroke may rarely be initial manifestation of eosinophilic granulomatosis with polyangiitis especially with late onset systemic features. Concurrent peripheral and central nervous system involvement associated with multisystemic features in late onset EGPA patients may have fulminant course therefore merit early suspicion and aggressive treatment.

## Data Availability

All the details of the case have been depicted in the manuscript.

## References

[1] Wolf J, Bergner R, Mutallib S, Buggle F, Grau AJ. Neurologic complications of Churg-Strauss syndrome--a prospective monocentric study. Eur J Neurol 2010;17:582–8. 20050889 10.1111/j.1468-1331.2009.02902.x

[2] Gioffredi A, Maritati F, Oliva E, Buzio C. Eosinophilic granulomatosis with polyangiitis: an overview. Front Immunol 2014;5:549. 25404930 10.3389/fimmu.2014.00549 PMC4217511

[3] Uchiyama M, Mitsuhashi Y, Yamazaki M, Tsuboi R. Elderly cases of Churg-Strauss syndrome: case report and review of Japanese cases. J Dermatol 2012;39:76–9. 22133207 10.1111/j.1346-8138.2011.01316.x

[4] Masi AT, Hunder GG, Lie JT, Michel BA, Bloch DA, Arend WP et al. The American College of Rheumatology 1990 criteria for the classification of Churg-Strauss syndrome (allergic granulomatosis and angiitis). Arthritis Rheum 1990;33:1094–100. 2202307 10.1002/art.1780330806

[5] Gomes I, Girão A, Gomes J, Rebelo O, Jesus-Ribeiro J. Neurological impact of eosinophilic granulomatosis with polyangiitis. Acta Neurol Belg 2022;122:123–8. 33905106 10.1007/s13760-021-01683-5

[6] Camara-Lemarroy CR, Infante-Valenzuela A, Villareal-Montemayor HJ, Soto-Rincon CA, Davila-Olalde JA, Villareal-Velazquez HJ. Eosinophilic granulomatosis with Polyangiitis presenting as acute polyneuropathy mimicking Guillain-Barre syndrome. Case Rep Neurol Med 2015;2015:981439. 26199772 10.1155/2015/981439 PMC4493297

[7] Oiwa H, Mokuda S, Matsubara T, Funaki M, Takeda I, Yamawaki T et al. Neurological complications in eosinophilic granulomatosis with polyangiitis (EGPA): the roles of history and physical examinations in the diagnosis of EGPA. Intern Med 2017;56:3003–8. 28924115 10.2169/internalmedicine.8457-16 PMC5726955

[8] Szczeklik W, Tutaj M, Sokołowska B, Mastalerz L, Miszalski-Jamka T, Dropiński J et al. Impaired cardiovascular autonomic nervous system function in patients with Churg-Strauss syndrome. Scand J Rheumatol 2011;40:304–7. 21366384 10.3109/03009742.2010.549500

